# Supplementary material for: Overexpression of GPX2 gene regulates the development of porcine preadipocytes and skeletal muscle cells through MAPK signaling pathway
Source: PLoS One. 2024 May 9;19(5):e0298827. doi: 10.1371/journal.pone.0298827 (PMC11081289; doi:10.1371/journal.pone.0298827)
Supplement: S1 Table — Primers for RT-PCR. (DOCX) [file pone.0298827.s001.docx]

**Supplemental Table S1. primer sequences of qRT-PCR**

| primer name | primer r sequence(5’-3’） | product size/bp |
| --- | --- | --- |
| susGPX2 1S | CTTCCTGGCTCCTCCTT | 150 |
| susGPX2 1AS | CTACCTTCTCCCCGTCC |  |
| sCyclinB S1 | AGGCAGATGGAAATGA | 293 |
| sCyclinB AS1 | ATAACCACAAGAAGGGA |  |
| sCdk2 F1 | CAAGGTGACGGGAGA | 232 |
| sCdk2 R2 | CAAGACCCGATGAGAAT |  |
| sP21 S1 | TGACATTTGGTCCCTG | 291 |
| sP21 AS1 | TTGGTTGCCTCCTTT |  |
| PPARγ F | AGGACTACCAAAGTGCCATCAAA | 142 |
| PPARγ R | GAGGCTTTATCCCCACAGACAC |  |
| ap2 F | GAGCACCATAACCTTAGATGGA | 121 |
| ap2 R | AAATTCTGGTAGCCGTGACA |  |
| FAS F | CGTGTGACCGCCATCTATATC | 125 |
| FAS R | ATACCACCAGAGACCGTTAT |  |
| CEBPα F | CGCGAGGAGGATGAAGCC | 168 |
| CEBPα R | CAGGTGCATGGTGGTCTGG |  |
| s-HSL-S1 | CGGTCTCCTCGGTTCCA | 110 |
| s-HSL-AS1 | GCACCCGCACTCCATA |  |
| s-ATGL-S1 | CATCATAACCCGCTTCGC | 165 |
| s-ATGL-AS1 | ATGGTGCTCTTGAGTTCGTAG |  |
| s-PLIN2-S2 | ATCAGCCAACAAACCA | 248 |
| s-PLIN2-R | GAATGCTTTTTCTACTCCACTGCTC |  |
| LPL F | GGAGAGAGGAAGGGAAAACAGAG | 150 |
| LPL R | AGACCGACCAATAAACTGCAAAG |  |
| PRKAG3 F | CTTGGGCTGGTGGAAGAGAA | 269 |
| PRKAG3 R | CCCACGAAGCTCTGCTTCTT |  |
| PKM F | TTAGCGGCAGCTTTGATAGTTC | 264 |
| PKM R | CACAATGACCACATCTCCCTTC |  |
| PFKM F | GTTGAACGACCTCCAGAAAGC | 300 |
| PFKM R | GGCGGACACTCAGGAATAAAA |  |
| MYH3 1S | GCCACGGATAGTGCCA | 311 |
| MYH3 1AS | CTTCTCGTAGACGGATTTG |  |
| MyoG F | AGGCTACGAGCGGACTGA | 230 |
| MyoG R | GCAGGGTGCTCCTCTTCA |  |
| MyoD F | AAGTCAACGAGGCCTTCGAG | 279 |
| MyoD R | GGGGGCCGCTATAATCCATC |  |
| Myostatin F | GATTATCACGCTACGACGGA | 269 |
| Myostatin R | CCTGGGTTCATGTCAAGTTTC |  |
| mGapdh F | AGGAGAGTGTTTCCTCGTCC | 60 |
| mGapdh R | TGCCGTGAGTGGAGTCATAC |  |
