# Supplementary figures and images for: Overexpression of GPX2 gene regulates the development of porcine preadipocytes and skeletal muscle cells through MAPK signaling pathway
Source: PLoS One. 2024 May 9;19(5):e0298827. doi: 10.1371/journal.pone.0298827 (PMC11081289; doi:10.1371/journal.pone.0298827)

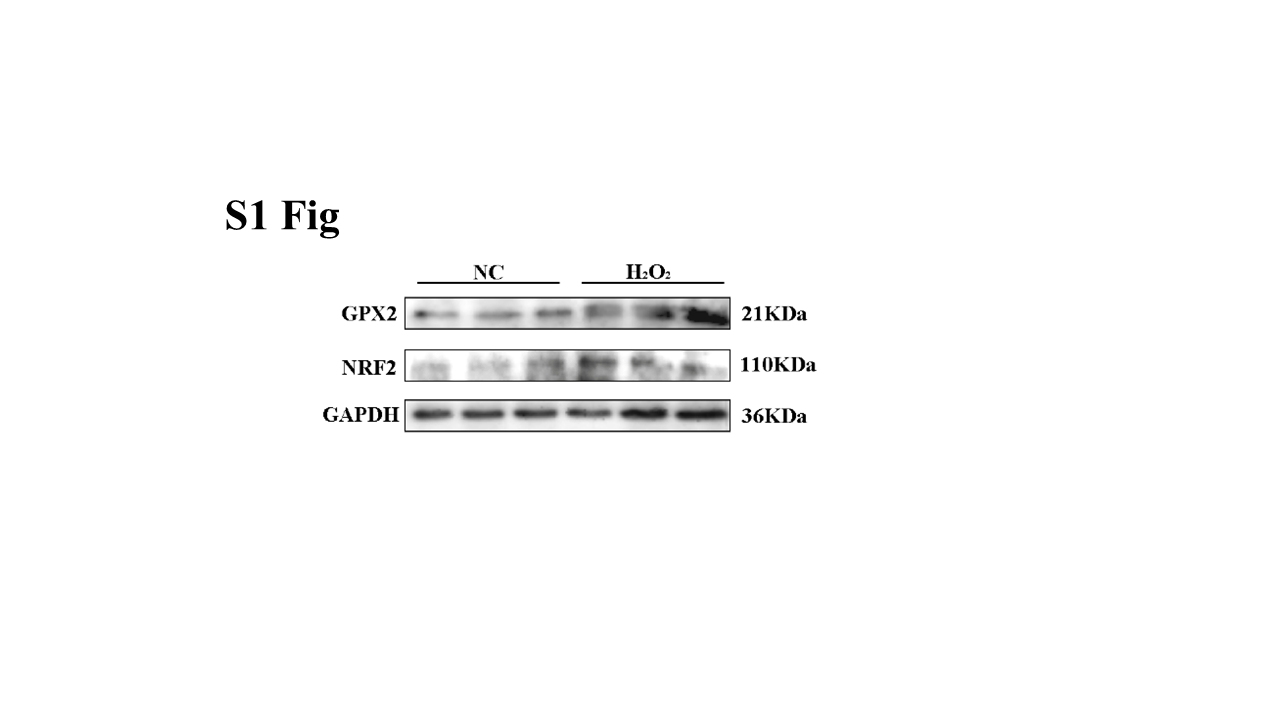

Supplement: S1 Fig — (TIF) [file pone.0298827.s002.tif]

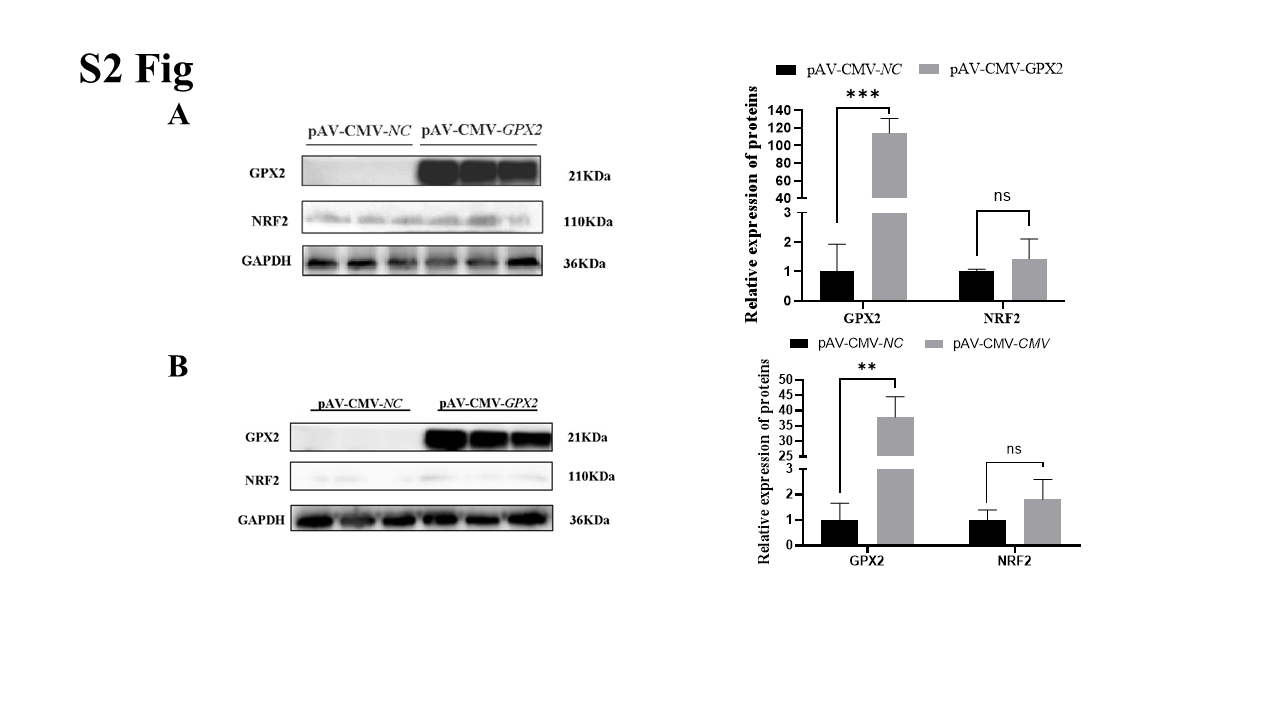

Supplement: S2 Fig — (A) Protein expressions and abundance analysis of GPX2 and NRF2 in the proliferative stage of porcine preadipocytes after overexpression of GPX2. (B) Protein expressions and abundance analysis of GPX2 and NRF2 in the differentiated stage of porcine preadipocytes. * p < 0.05, ** p < 0.01, *** p < 0.001, ns indicates not significant. (TIF) [file pone.0298827.s003.tif]

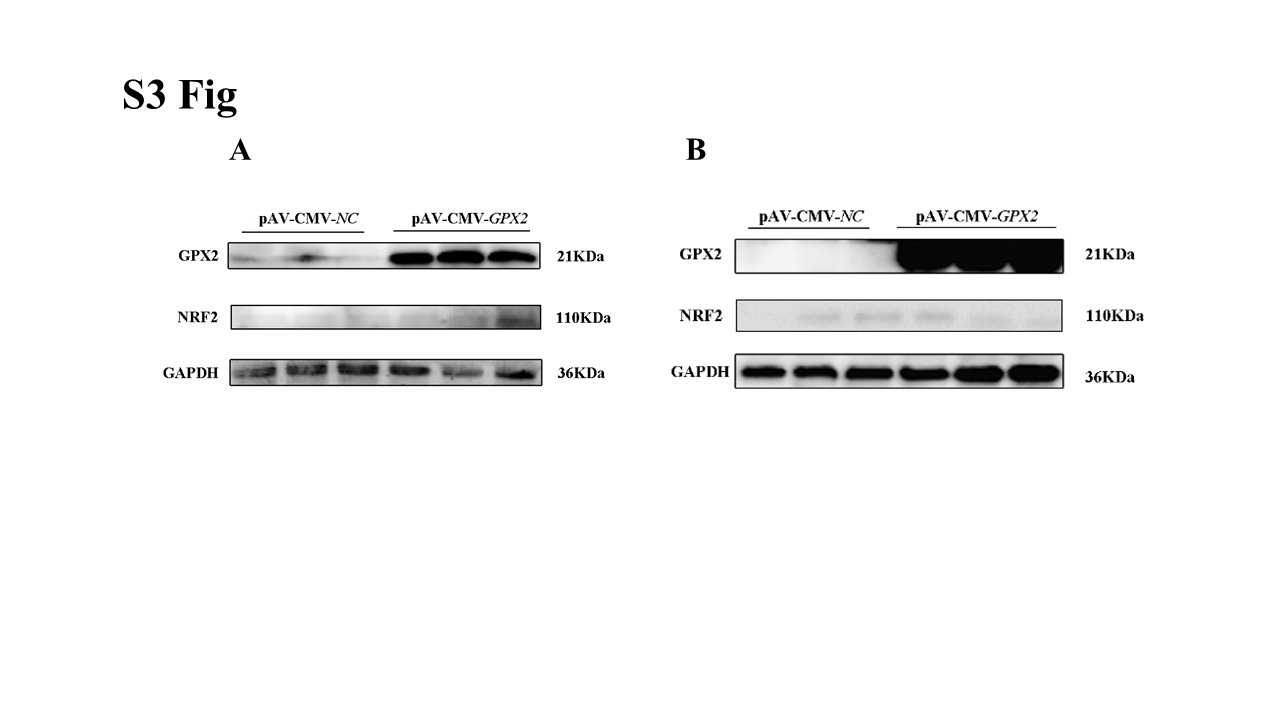

Supplement: S3 Fig — (A) Protein expressions of GPX2 and NRF2 in the proliferative stage of porcine skeletal muscle cells after overexpression of GPX2. (B) Protein expressions of GPX2 and NRF2 in the differentiated stage of porcine skeletal muscle cells. (TIF) [file pone.0298827.s004.tif]
