# Supplementary material for: Overexpression of GPX2 gene regulates the development of porcine preadipocytes and skeletal muscle cells through MAPK signaling pathway
Source: PLoS One. 2024 May 9;19(5):e0298827. doi: 10.1371/journal.pone.0298827 (PMC11081289; doi:10.1371/journal.pone.0298827)

Original Images for Western Blot

Figure 1

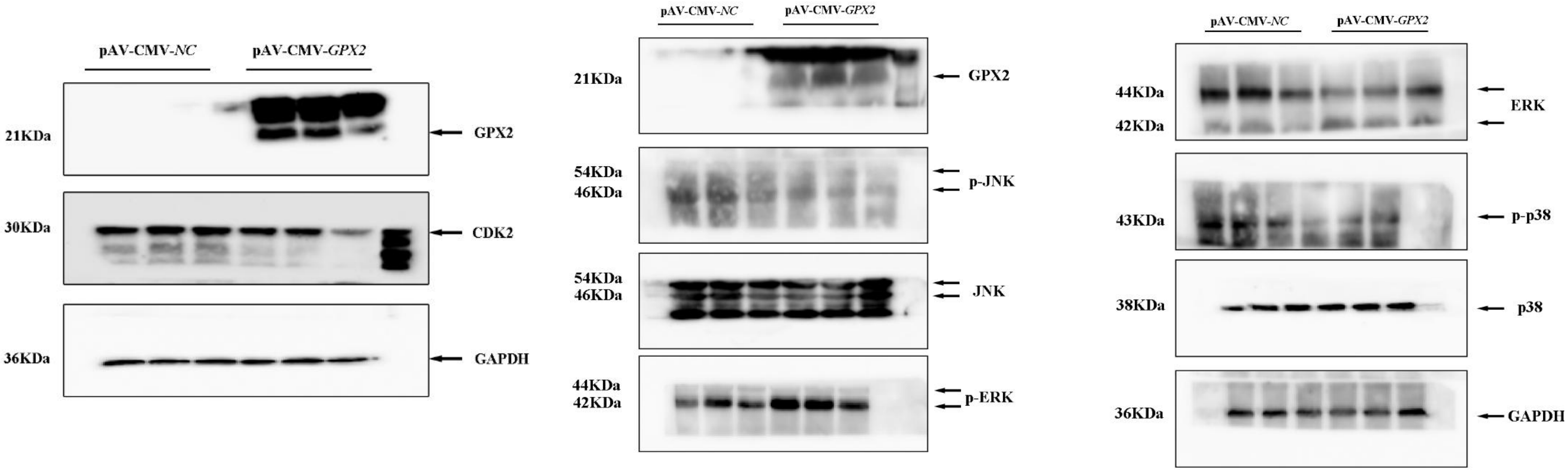

Figure 2

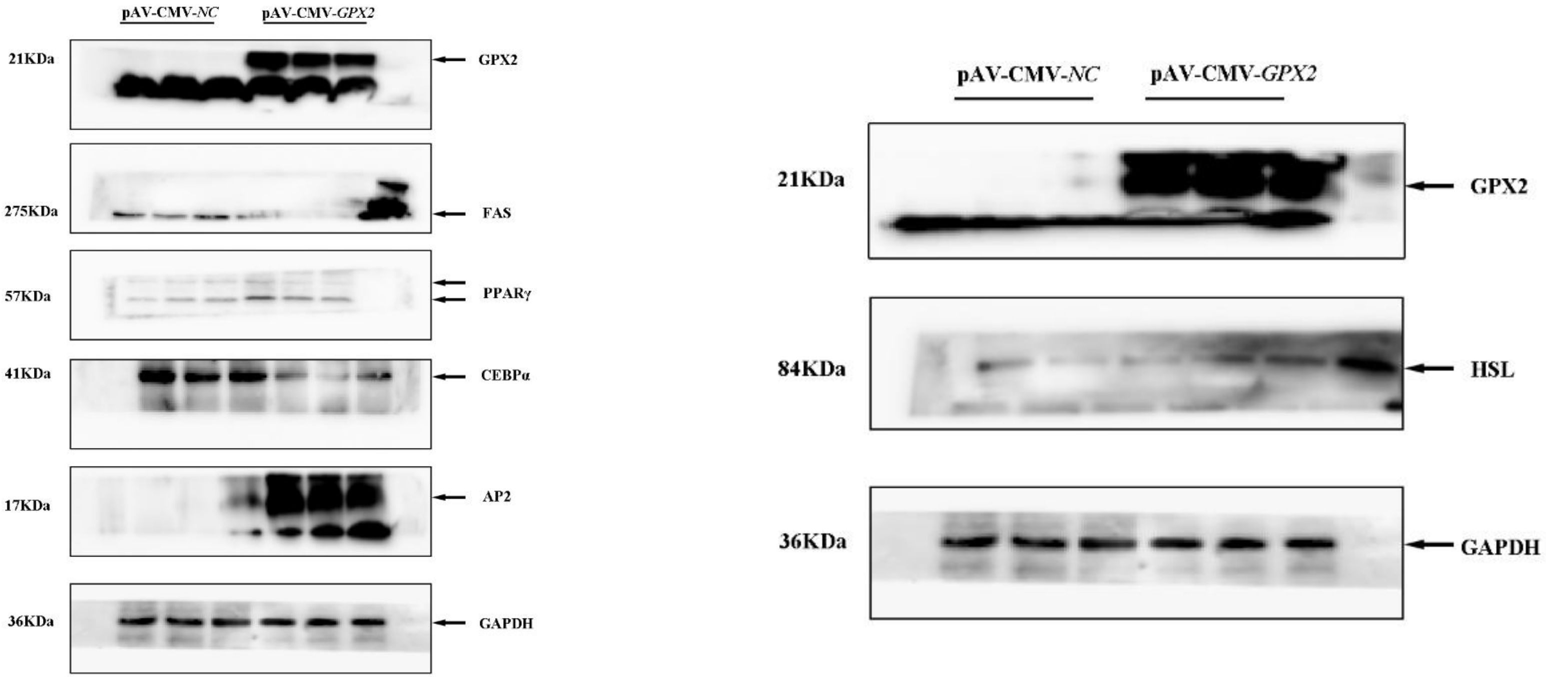

Figure 3

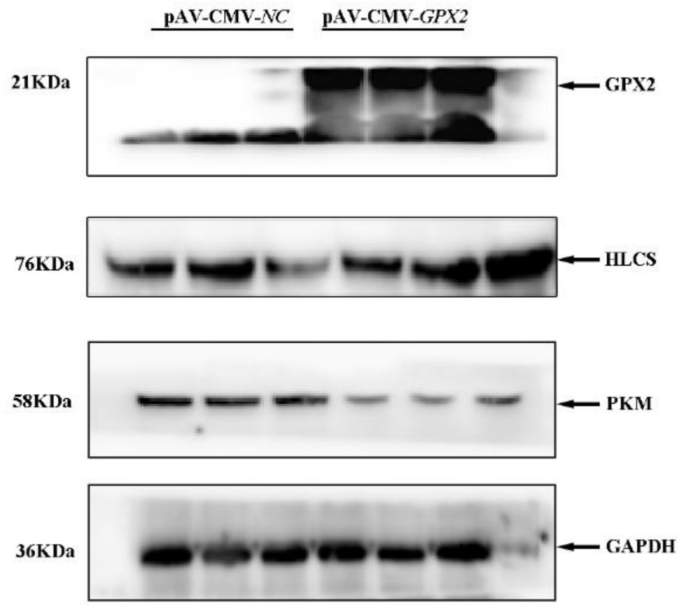

Figure 4

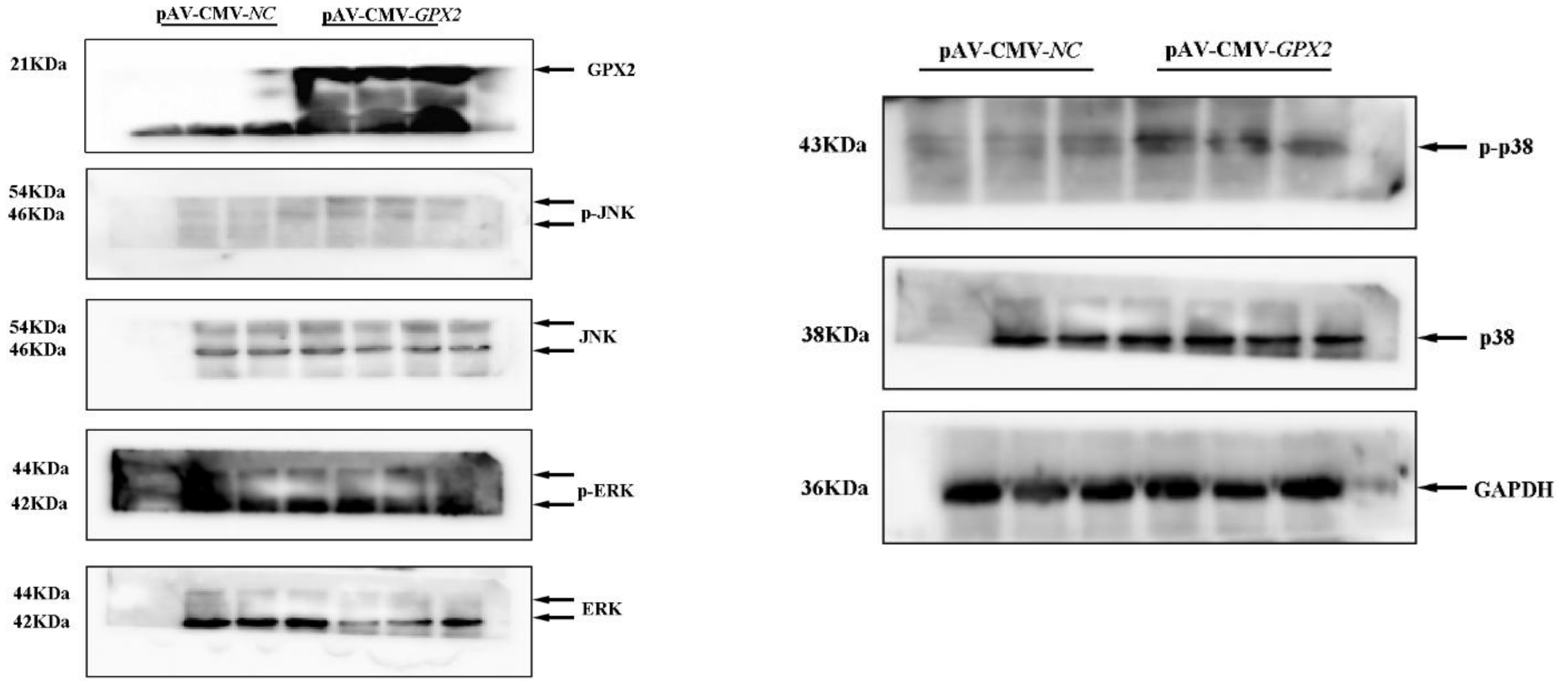

Figure 5

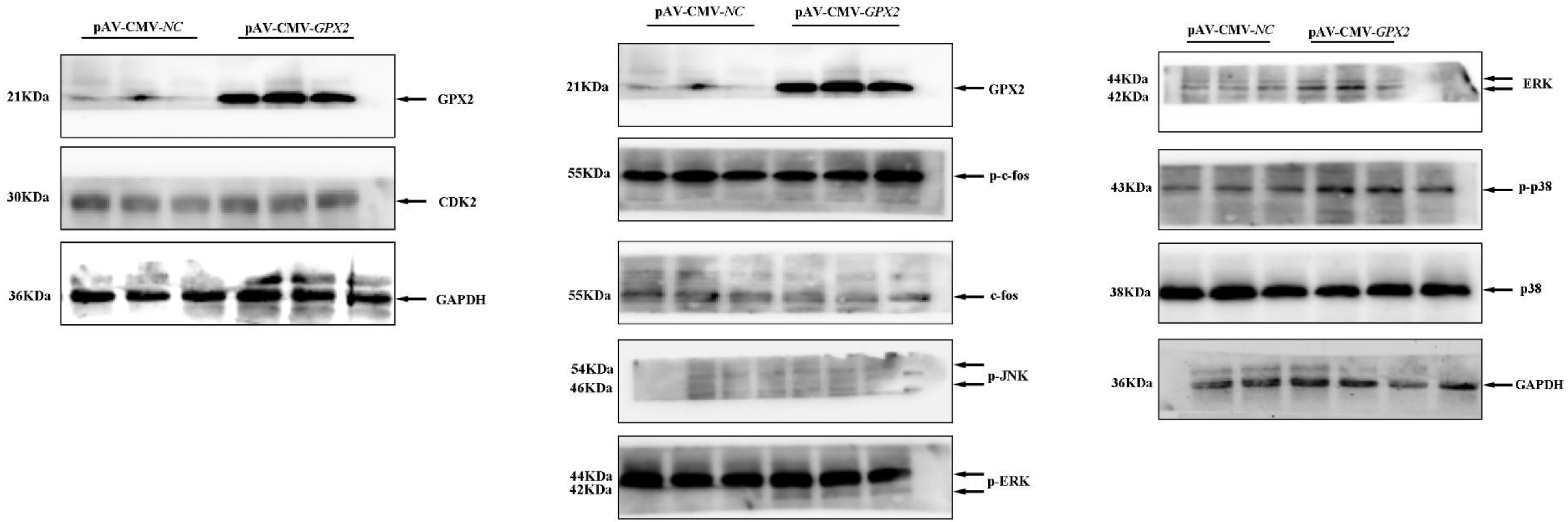

Figure 6

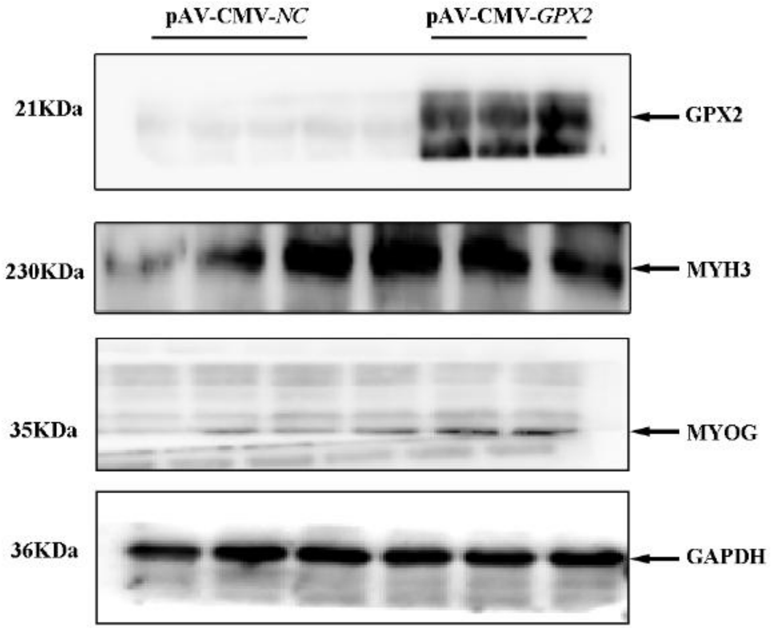

Figure 7

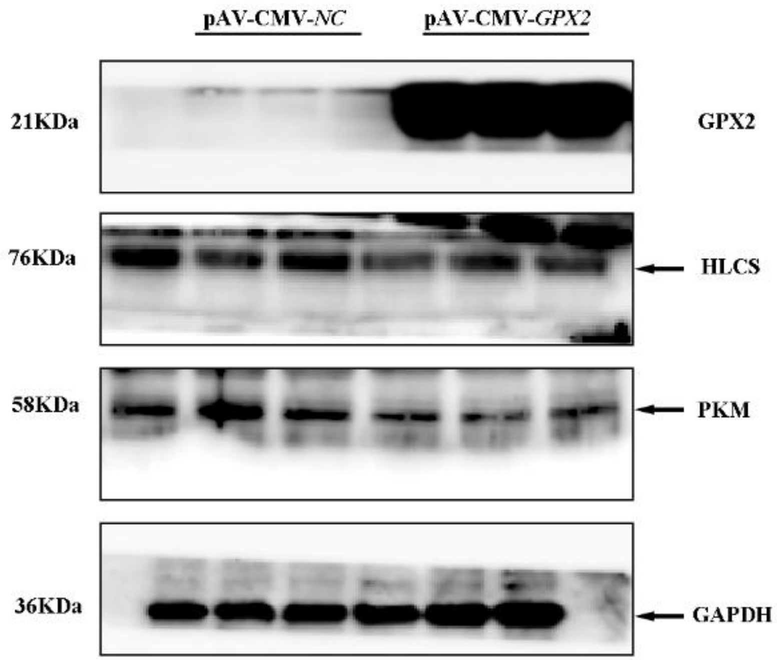

Figure 8

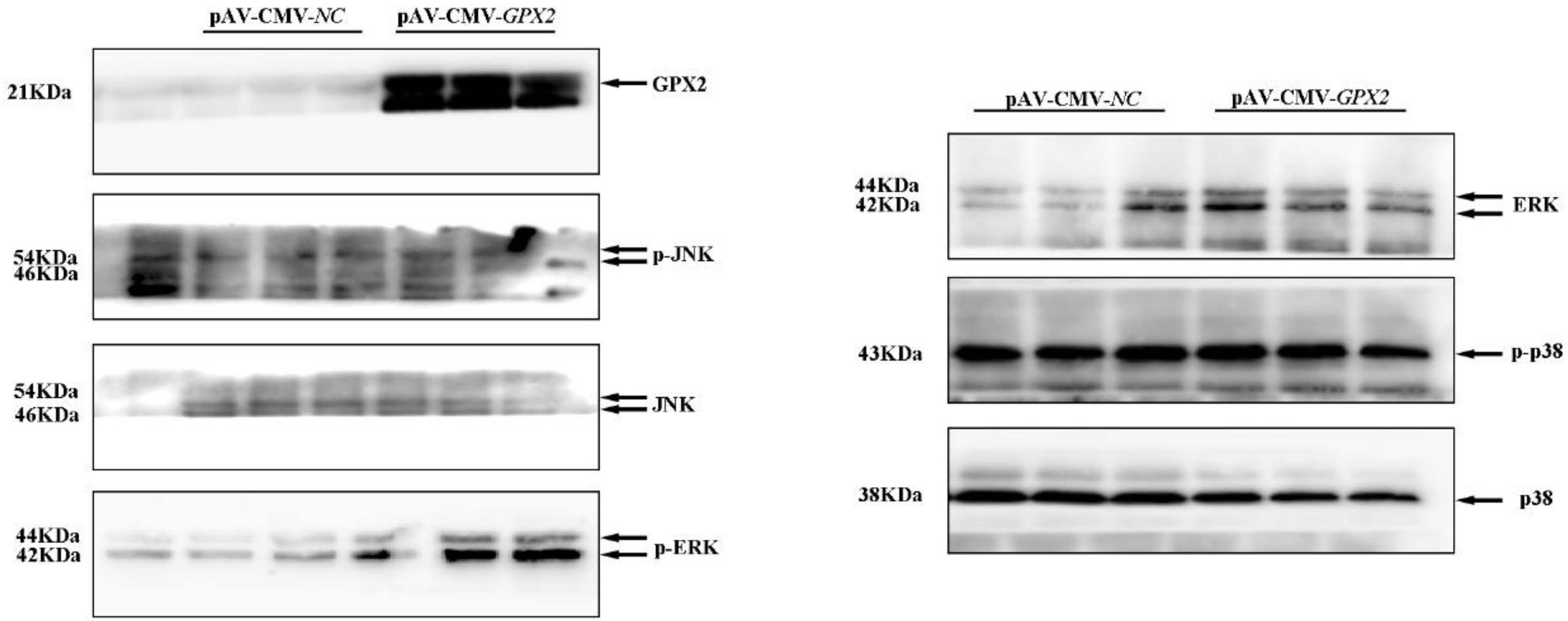

S1 Fig

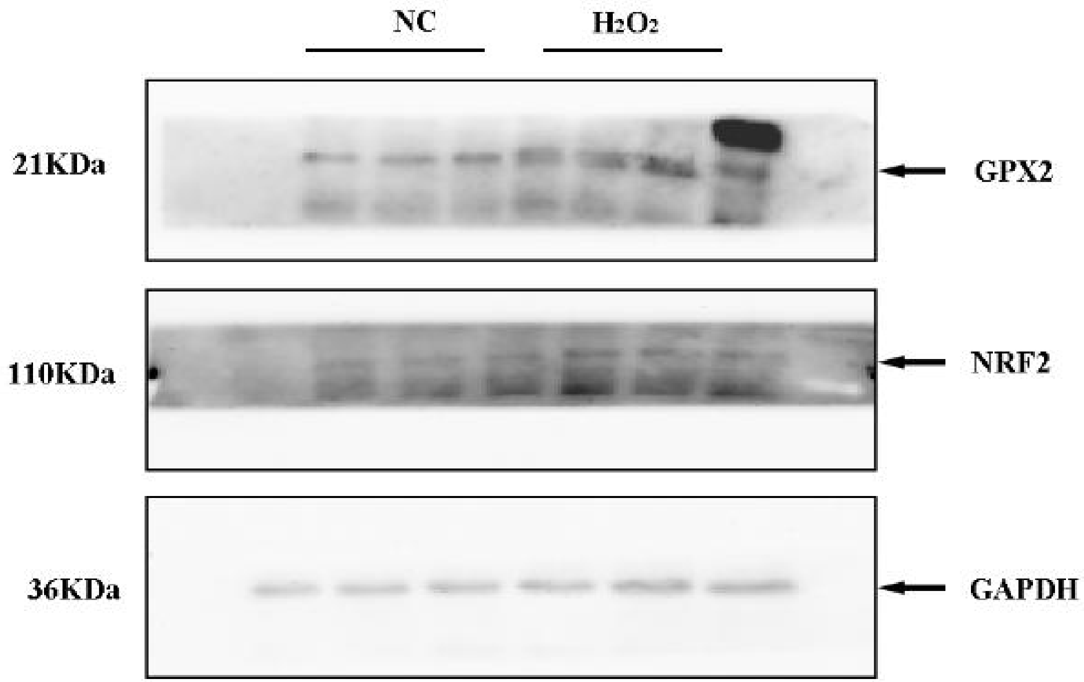

A

B

S2 Fig

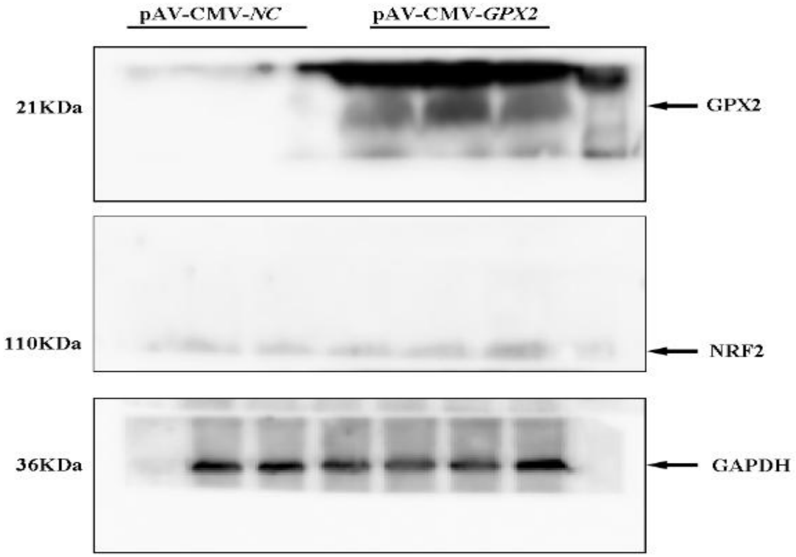

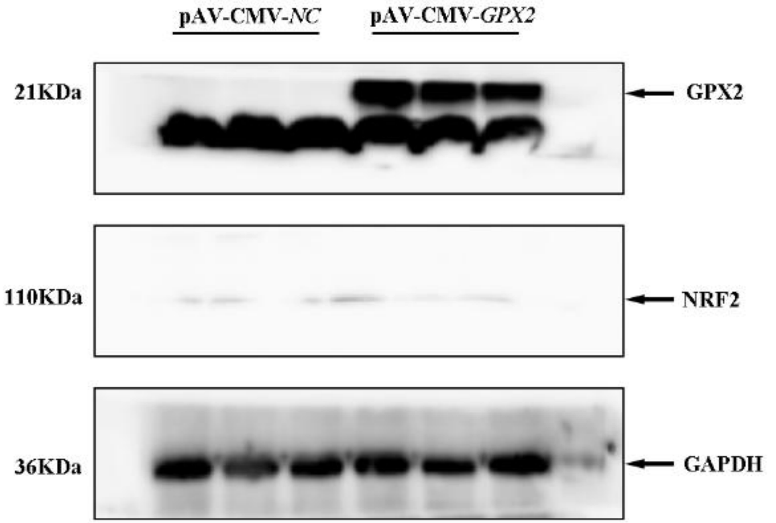

S3 Fig

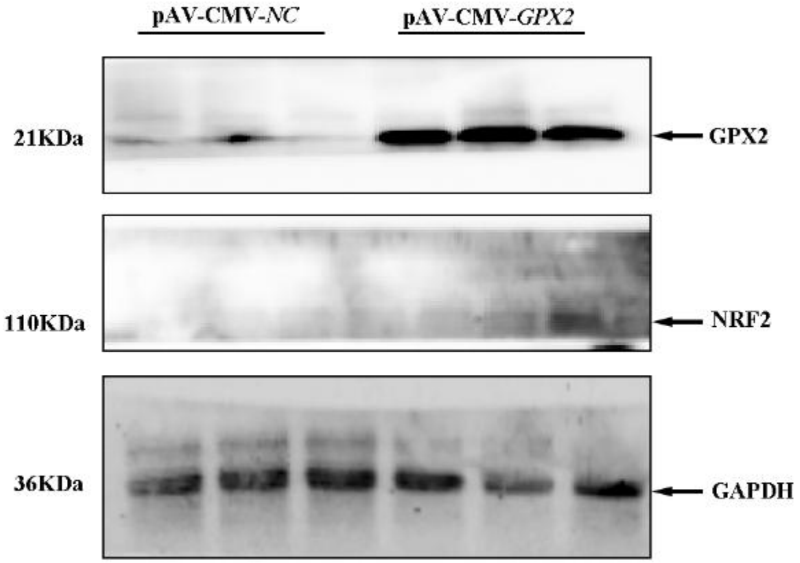

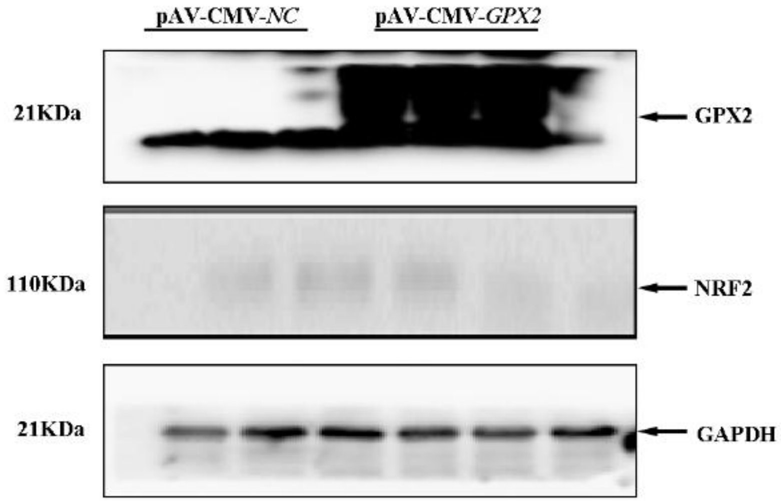

Supplement: S1 File — (PDF) [file pone.0298827.s005.pdf]
