# Supplementary material for: Overexpression of GPX2 gene regulates the development of porcine preadipocytes and skeletal muscle cells through MAPK signaling pathway
Source: PLoS One. 2024 May 9;19(5):e0298827. doi: 10.1371/journal.pone.0298827 (PMC11081289; doi:10.1371/journal.pone.0298827)
Supplement: Attachment — Submitted filename: Response to Reviewers.docx [file pone.0298827.s006.docx]

Dear reviewer：

Thank you very much for your timely reply and very professional comments and useful suggestions, which has significantly raised the quality of the manuscript. We have given careful consideration to every comments and suggestions. The following is a peer-to-peer response and revision to the various comments and suggestion.

**Questions and suggestion 1:** Why did you choose GPX2 not GPX1, GPX3 or GPX4? In other words, what is the difference between GXP1 and the other GPXs? Please clarify this in the introduction. Did you check the expression GPXs in pigs? Is GPX2 expression greater or lower than the other GPXs? Also, did you check the expression of the other GPXs when you overexpressed GPX2 in your cells?

**Author’s answer 1:** Thank you very much for such a professional question. Previous studies have reported that GPX2 is an important candidate gene for RFI traits. The authors also found that the type of SNP mutation in the GPX2 gene was significantly associated with backfat thickness and average daily gain in pigs (Pu et al., 2022). These contents are also added to the introduction of this article according to the experts' suggestions. GPX2 is a member of gastrointestinal GPX, but numerous studies have found that it plays an important role in muscle and adipose tissue. The pig which have the different genotypes of GPX2 have the difference expression of GPX2 in muscle and backfat tissues. It is moderately expressed in muscle and adipose tissue. The specific results are shown below (Pu et al., 2022). For this reason, this study mainly studied the function of GPX2.

(Results from Pu et al., 2022)

**Questions and suggestion 2:** Since GPX2 plays critical role in regulating oxidative stress, why did you overexpress it in adipocytes and myocytes not liver? Liver is one of the major sites for ROS production. Have you checked the expression of GPX2 in different tissues in pigs?

**Author’s answer 2:** Our previous results showed that GPX2 was more highly expressed in adipose tissue, muscle tissue, lungs and ileum of pigs, while there was no significant difference in the liver(Pu et al., 2022). From the aspect of animal nutrition, GPX2 has been partially reported in intestinal. But it has not been reported in adipocytes and myocytes of pig. So that's why we did this study.

**Questions and suggestion 3:** Why did you choose overexpress of GPX2 rather than knockout or knockdown it?

**Author’s answer 3:** The reviewer's question is so professional. I've also always wanted to study the effects of knockdown the GPX2 gene. Because the CDs regions of the GPX2 was very short (just over 500 bp). We also consulted a number of biological companies, but they were unable to design suitable siRNA fragments, so this part of the experiment could not be carried out.

**Questions and suggestion 4:** Is there enough evidence suggests that selenium supplementation can increase the expression of GPX2? If not the conclusion about "selenium as a nutritional supplement to promote human subcutaneous fat reduction and muscle growth" should be revised.

**Author’s answer 4:** The reviewer's question are very precise. This article does not study the functions of selenium, so the text in the article is not rigorous. This part of the content will be deleted.

**Questions and suggestion 5:** Make sure the format is consistent. For example, Line 88: "Porcine skeletal muscle cells culture and myogenic differentiation" is not bold.

**Author’s answer 5:** I am very sorry for such an inappropriate formatting error. I will correct this error and further check the article carefully to avoid relevant formatting errors.

**Questions and suggestion 6:** I feel something is missed between oxidative stress and lipid metabolism. In the introduction you mentioned GPX2 plays an important role in regulating oxidative stress, but how does this lead you to determine whether overexpression of GPX2 may change lipid metabolism? Please make the logic more clear and then readers can understand your story easier.

**Author’s answer 6:** Thanks for the reviewer's advice. This part of the content is really not clear. I will revise it in the article. The SNPs of GPX2 gene was found to be significantly associated with backfat thickness in Duroc pigs. Because GPX2 is a gene associated with oxidative stress, we studied the effects of overexpression of GPX2 on oxidative stress, lipid metabolism and glycolysis. In the supplement figure of this paper, it is listed that overexpression of GPX2 gene does not affect the expression of NRF2 protein in adipocytes and myocytes of pig. Combined with the results in the text, we concluded that GPX2 gene promotes lipid degradation by inhibiting lipid deposition in adipocytes, and at the same time leads to abnormal of glycolysis.

The logic in the article is indeed not clear enough, and we will further modify the introduction and discussion.

**Questions and suggestion 7:** Based on your results, it seems that GXP2 plays different roles in different type of cells. Therefore, you should make careful conclusions that increased expression of GPX2 might be good for human health. Because overexpression of GPX2 in other cells such as hepatocyes or immune cells might not be good for human health. More research is required to further demonstrate the role of GPX2 for overall health in an animal or human.

**Author’s answer 7:** I strongly agree with the opinion of the reviewer. The description that GPX2 overexpression promotes human health is really inadequate. The authors agreed that it would be more appropriate for this section to be deleted.

**Questions and suggestion 8:** Your results indicate that oxidative stress can increase GPX2. Besides, overexpression of GPX2 seems to be good for health since it inhibit adipocyte proliferation and promotes muscle cell growth. Therefore, do you think mild oxidative stress might be good for health?

**Author’s answer 8:** In fact, GPX2 expression increases after oxidative stress, when the body activates repair mechanisms. GPX2 belongs to the family of glutathione peroxidases, which have an antioxidant effect and reduce oxidative stress. Oxidative stress is known to be bad for animal growth. Therefore, we can think from another angle, through genetic methods, find the SNPs that can promote GPX2 expression, and achieve the selection of individuals with high GPX2 genes, which is the previous research results of our team(Pu et al., 2016). We will also revise this part in the discussion.

Thanks again to the reviewer for their professional suggestions and comments. We have revised the article accordingly. If the editorial department has any questions, please do not hesitate to contact us directly.

Have a nice day!

Sincerely

Lei Pu

E-mail: pulei87@126.com

*Tianjin Key Laboratory of Agricultural Animal Breeding and Healthy Husbandry, College of Animal Science and Veterinary Medicine, Tianjin Agricultural University, Tianjin 300384, China.*

**Reference**

Pu L, Luo Y, Wen Z, Dai Y, Zheng C, Zhu X, et al. GPX2 Gene Affects Feed Efficiency of Pigs by Inhibiting Fat Deposition and Promoting Muscle Development. Animals (Basel). 2022;12(24). Epub 2022/12/24. doi: 10.3390/ani12243528. PubMed PMID: 36552449; PubMed Central PMCID: PMCPMC9774625.
