# Supplementary material for: Overexpression of GPX2 gene regulates the development of porcine preadipocytes and skeletal muscle cells through MAPK signaling pathway
Source: PLoS One. 2024 May 9;19(5):e0298827. doi: 10.1371/journal.pone.0298827 (PMC11081289; doi:10.1371/journal.pone.0298827)
Supplement: Attachment — Submitted filename: Response to Reviewers.docx [file pone.0298827.s007.docx]

Dear reviewer：

Thank you very much for your reply about professional comments and helpful suggestions, which have significantly raised the quality of the manuscript.

We have studied the editor’s requirements. We have carefully modified the format errors of the references. We have also inquired the rejection and withdrawal references mentioned by the editor, and All references have no problem. The editor also suggested the paper English language needs to be polished. Need find native English speaker to revise the manuscript and make it more readable. We have asked an native English speaker who is animal science professionals to revise this article. After modification, the language of the article has been standardized a lot.

Thanks again to the reviewer for their suggestions and comments. We have revised the article accordingly. If the editorial department has any questions, please do not hesitate to contact us directly.

Have a nice day!

Sincerely

Lei Pu

E-mail: pulei87@126.com

*Tianjin Key Laboratory of Agricultural Animal Breeding and Healthy Husbandry, College of Animal Science and Veterinary Medicine, Tianjin Agricultural University, Tianjin 300384, China.*
