# Supplementary material for: Overexpression of GPX2 gene regulates the development of porcine preadipocytes and skeletal muscle cells through MAPK signaling pathway
Source: PLoS One. 2024 May 9;19(5):e0298827. doi: 10.1371/journal.pone.0298827 (PMC11081289; doi:10.1371/journal.pone.0298827)
Supplement: Attachment — Submitted filename: Response to Reviewers.docx [file pone.0298827.s008.docx]

Dear reviewer：

Thank you sincerely for your response regarding the professional comments and helpful suggestions, which have significantly enhanced the manuscript's quality. We have thoroughly considered each comment and suggestion. The subsequent peer-to-peer response and revision address the comments and suggestions in detail.

**Questions and suggestion 1:** Western blot: I noticed that repeated showing the western blot of GPX2 overexpression. I assume that you used the same cell lysates blotted many proteins. It is fine and you don't need to showing the same GPX2 overexpressed bands all the time.

**Author’s answer 1:** The reviewer's question are very precise. We used the same cell lysates to blot many proteins. But to make it easier to distinguish, we replaced the same *GPX2* overexpressed band.

**Questions and suggestion 2:** IF staining: Figure 6 could you please zoom in the IF staining and find more representative images to show the location of nuclei? Based on the images you provided, it is hard for the reviewer to see the difference.

**Author’s answer 2:** The reviewer's question is so professional. We analyzed 12 staining images about each of the NC group and *GPX2* overexpression group, and observed overexpressing *GPX2* had an increased effect on the differentiation index and fusion index in porcine skeletal muscle cells, but no significant difference. We replaced the images with clearer images, but the conclusion did not change.

**Questions and suggestion 3:** The resolution of your figure is pretty low. Figures look blur. Please fix it.

**Author’s answer 3:** We found the corresponding original figure and modified the figure format to ensure that the resolution of the figure met the requirements.

Thanks again to the reviewer for their suggestions and comments. We have revised the article accordingly. If the editorial department has any questions, please do not hesitate to contact us directly.

Have a nice day!

Sincerely

Lei Pu

E-mail: pulei87@126.com

*Tianjin Key Laboratory of Agricultural Animal Breeding and Healthy Husbandry, College of Animal Science and Veterinary Medicine, Tianjin Agricultural University, Tianjin 300384, China.*

**Fig 1.** **Overexpression of *GPX2* inhibits proliferation of porcine preadipocytes.**

**Fig 2.** **Overexpression of *GPX2* inhibits adipogenic differentiation and promoted the lipid degradation of porcine preadipocytes**.

**Fig 3.** **Overexpression of *GPX2* promotes glycolysis-related genes expression of porcine preadipocytes**.

**Fig 4.** **The change of MAPK signaling pathway after overexpression with *GPX2***.

**Fig 5.** **Overexpression of *GPX2* promotes proliferation of porcine skeletal muscle cells**.

**Fig 6.** **Overexpression of *GPX2* promotes myoblastic differentiation of porcine skeletal muscle cells**.

**Fig 7.** **Overexpression of *GPX2* inhibits glycolysis of porcine skeletal muscle cells**.**Fig 8.** **The changes of MAPK signaling pathway after overexpression of *GPX2***.

**Fig 9.** **Hypothesis schematic models illustrating the *GPX2* affects the proliferation and differentiation of porcine preadipocytes and skeletal muscle cells by regulating MAPK pathways.** ↑ indicates expression up-regulation, ↓ indicates expression down-regulation.
